# Exploring Self-Reported Symptoms for Developing and Evaluating Digital Symptom Checkers for Polycystic Ovarian Syndrome, Endometriosis, and Uterine Fibroids: Exploratory Survey Study

**DOI:** 10.2196/65469

**Published:** 2024-12-12

**Authors:** Aidan P Wickham, Yella Hewings-Martin, Frederick GB Goddard, Allison K Rodgers, Adam C Cunningham, Carley Prentice, Octavia Wilks, Yusuf C Kaplan, Andrei Marhol, András Meczner, Heorhi Stsefanovich, Anna Klepchukova, Liudmila Zhaunova

**Affiliations:** 1 Flo Health UK Limited London United Kingdom; 2 Fertility Centers of Illinois Vernon Hills, IL United States

**Keywords:** polycystic ovary syndrome, PCOS, self-assessment, self-reported, endometriosis, uterine fibroids, symptoms, digital symptom checker, women's health, gynecological conditions, reproductive health

## Abstract

**Background:**

Reproductive health conditions such as polycystic ovary syndrome (PCOS), endometriosis, and uterine fibroids pose a significant burden to people who menstruate, health care systems, and economies. Despite clinical guidelines for each condition, prolonged delays in diagnosis are commonplace, resulting in an increase to health care costs and risk of health complications. Symptom checker apps have the potential to significantly reduce time to diagnosis by providing users with health information and tools to better understand their symptoms.

**Objective:**

This study aims to study the prevalence and predictive importance of self-reported symptoms of PCOS, endometriosis, and uterine fibroids, and to explore the efficacy of 3 symptom checkers (developed by Flo Health UK Limited) that use self-reported symptoms when screening for each condition.

**Methods:**

Flo’s symptom checkers were transcribed into separate web-based surveys for PCOS, endometriosis, and uterine fibroids, asking respondents their diagnostic history for each condition. Participants were aged 18 years or older, female, and living in the United States. Participants either had a confirmed diagnosis (condition-positive) and reported symptoms retrospectively as experienced at the time of diagnosis, or they had not been examined for the condition (condition-negative) and reported their current symptoms as experienced at the time of surveying. Symptom prevalence was calculated for each condition based on the surveys. Least absolute shrinkage and selection operator regression was used to identify key symptoms for predicting each condition. Participants’ symptoms were processed by Flo’s 3 single-condition symptom checkers, and accuracy was assessed by comparing the symptom checker output with the participant’s condition designation.

**Results:**

A total of 1317 participants were included with 418, 476, and 423 in the PCOS, endometriosis, and uterine fibroids groups, respectively. The most prevalent symptoms for PCOS were fatigue (92%), feeling anxious (87%), BMI over 25 (84%); for endometriosis: very regular lower abdominal pain (89%), fatigue (85%), and referred lower back pain (80%); for uterine fibroids: fatigue (76%), bloating (69%), and changing sanitary protection often (68%). Symptoms of anovulation and amenorrhea (long periods, irregular cycles, and absent periods), and hyperandrogenism (excess hair on chin and abdomen, scalp hair loss, and BMI over 25) were identified as the most predictive symptoms for PCOS, while symptoms related to abdominal pain and the effect pain has on life, bleeding, and fertility complications were among the most predictive symptoms for both endometriosis and uterine fibroids. Symptom checker accuracy was 78%, 73%, and 75% for PCOS, endometriosis, and uterine fibroids, respectively.

**Conclusions:**

This exploratory study characterizes self-reported symptomatology and identifies the key predictive symptoms for 3 reproductive conditions. The Flo symptom checkers were evaluated using real, self-reported symptoms and demonstrated high levels of accuracy.

## Introduction

### Background

Polycystic ovary syndrome (PCOS), endometriosis, and uterine fibroids are among the most prevalent reproductive health conditions, collectively impacting millions of women and people who menstruate globally. Prevalences of PCOS and endometriosis range from 4%-20% and 5%-10%, respectively [1-4]. For uterine fibroids, prevalence of diagnosis is approximately 10% across the female population [5], and importantly, the condition has a high estimated cumulative incidence by onset of menopause, occurring in over 70% of women [6,7].

Despite the symptoms of these conditions being well-established in medical guidelines, diagnostic delays are common, with patients enduring between 2 and 12 years from the onset of their symptoms before they receive a formal diagnosis [8-10]. These delays could be due to complexities in diagnostic procedure [11], controversy in diagnostic evaluation [12], or affected persons having low knowledge of reproductive conditions and their symptoms [13].

Self-assessed, self-reported symptoms are known to be an effective means of identifying those who need medical attention [14], and their severity has been shown to correlate with diagnosis [15,16]. Better understanding of how those affected by these reproductive conditions self-assess and self-report their symptoms is vital, as left untreated these conditions can lead to worsening of symptoms, infertility, and further health complications [13,17-19]. People are increasingly trusting mobile apps and the internet as a source of health information [20-22] and intervention to assist with explaining or recognizing their symptoms [23,24]. “Symptom checkers” are a digital health tool that allow users to match their symptoms against those of health conditions. Symptom checkers cannot provide diagnosis to the user, but they do hold the potential to facilitate screening of reproductive conditions at scale, and therefore, rigorous validation and proof of their accuracy is essential [25]. A validation process that uses self-reported symptoms from real people will help to understand how a symptom checker will perform once deployed.

### Flo App and Symptom Checker Development

Flo (by Flo Health UK Limited) is a period-tracking mobile app [26] allowing users to log their menstruation, ovulation, contraception, symptoms, and other physical and emotion-based metrics. Flo has developed 3 single-condition symptom checkers for PCOS, endometriosis, and uterine fibroids [27] that are based on current guidelines (Monash, AAFP, and ESHRE) [28-30]. Interactions with these are either user-led or triggered by symptoms or events logged in the app (eg, detection of cycle irregularity). The symptom checkers ask users conversation-like questions to gather information on symptoms, medical, and family history related to each condition. The symptom checkers are not diagnostic tools; the user receives an outcome of “significant match,” “low match,” or “no match,” and an informative summary of their symptomatology that can be used to facilitate conversations with their health care provider should they wish to seek subsequent medical investigations. The symptom checkers use a cumulative total of present symptoms over a threshold to determine the condition-match output the user receives.

### Aim

This exploratory study sought to estimate self-reported symptomatology of PCOS, endometriosis, and uterine fibroids, and investigate which symptoms were most predictive of each condition. We also explored the potential accuracy of three single-condition symptom checkers when assessing self-reported symptoms.

## Methods

### Self-Reported Symptom Data Collection

#### Participants

Subscribers to the paid survey platform “Prolific” were invited to take part in a study related to female reproductive health from September to December 2023. Participants were included if they consented, were aged 18 years or older, female, and living in the United States. Due to the relatively low prevalence of each condition, participants were prescreened to ensure there were enough participants with a condition diagnosis included in the study. Participants were invited to complete a survey related to either PCOS, endometriosis, or uterine fibroids. Informed electronic consent was obtained from all participants at the start of the survey. 

#### Surveys

The questions from each single-condition symptom checker were transcribed into 3 separate surveys. The surveys comprised the following sections: (1) examination history for the condition in question, (2) medical history relevant to the condition, and (3) the questions from each symptom checker. In addition, the PCOS symptom checker contains images which show examples of hyperandrogenism symptoms, which was reflected in the PCOS survey.

Respondents who indicated they had a positive diagnosis for PCOS, endometriosis, or uterine fibroids were asked to answer symptom questions retrospectively. Respondents who did not have a diagnosis for one of these 3 conditions answered the surveys about their current symptoms.

#### Case Designation

Condition-positive (CP) cases were designated as those participants who had received a positive diagnosis for PCOS, endometriosis, or uterine fibroids from their doctor. It is possible CP participants were multimorbid with 2 or more of the conditions investigated in this study.

Condition-negative (CN) cases were designated as those participants who responded they had not been examined by a doctor for the condition. We did not use participants with a confirmed negative diagnosis (ie, examined for the condition and told they do not have it) as our CN cases, as their visits to a doctor might indicate potential symptoms for other conditions, making them unsuitable as control cases. We assumed that most undiagnosed, unexamined participants will be negative for the condition; it is possible that CN cases may unknowingly have the condition, but this bias is expected to be relatively small due to the symptomatic prevalence of each condition within reproductive age being approximately 10% or less [1,3,5,31].

### Analysis

#### Participant Refinement

CP participants were excluded from the analysis if they were outside the ages of 18 and 45 years at the time of their diagnosis (calculated from their age and year of diagnosis, provided in their survey responses), while CN participants were excluded if they were outside the ages of 18 and 45 years at the time of survey completion. As CN participants answered questions about their current symptom status (as opposed to their retrospective symptom status), CN participants were excluded if they were currently pregnant, had recently given birth, or were currently taking hormonal contraceptives, in case these elements interfered with their symptomatology. PCOS-negative participants were also excluded if they had recently had a miscarriage due to the effect this can have on menstrual cycle regularity. Endometriosis-negative and uterine fibroids-negative participants were not excluded on this criterion, as miscarriage has been shown to be associated with both conditions [32-34].

By definition, our symptom checkers can only detect symptomatic cases of each condition. Due to the high-rate of uterine fibroids cases being asymptomatic [35], we asked uterine fibroids-positive participants if they were symptomatic or not at the time of their diagnosis. Participants who responded “I was asymptomatic (had no symptoms)” were excluded from the analysis. Participants with PCOS and endometriosis were not asked if they were asymptomatic at the time of their diagnosis, and so our analysis may include some asymptomatic cases of these 2 conditions. Participants who did not complete the entire survey were removed from the analysis.

#### Symptom Presence

The presence or absence of a symptom was recorded for each participant based on their answer to each survey question. Neutral responses to questions such as “I don’t know” or “I’d prefer not to answer” were recorded as an absent symptom.

#### Statistical Analysis

Symptom prevalence was calculated relative to the number of individuals in each CP or CN group; for symptoms related to fertility or pregnancy, symptom prevalence was calculated relative to those participants who had indicated they either had previously tried or were currently trying to conceive. Chi-square test without correction was used to test statistical difference in symptom prevalence between CP and CN groups.

To select the most important symptoms for detecting a CP outcome from our self-reported symptom dataset, we used the use of least absolute shrinkage and selection operator (LASSO) regression. The LASSO regression provided feature importance scores for the most predictive symptoms of the CP outcome, from which we present the top 10 symptoms for each condition.

Participants’ symptoms were processed by each symptom checker’s cumulative algorithm and the outcome was recorded as either a “significant match (symptoms suggestive of the condition) or a “low or no match” (symptoms not suggestive enough of the condition). To produce accuracy metrics, “significant match” was considered equivalent to CP, and “low or no match” was considered equivalent to CN. Accuracy was calculated as the sum of correctly identified CP and CN cases divided by the total number of cases for each condition.

#### Sensitivity Analysis

A sensitivity analysis investigated self-reported symptom prevalence in participants with a confirmed-negative diagnosis (examined for the condition and told they do not have it). We also tested the performance of the Flo symptom checkers using this confirmed-negative group.

### Ethical Considerations

This study and protocol were approved by the Independent Ethical Review Board: WIRB-Copernicus Group Institutional Review Board (IRB number 20231821). All participants completed an electronic, informed consent form, agreeing to their health and personal data being processed. Participants were informed their responses would be deidentified and all published results would be aggregated. Participants were compensated approximately $11.62/hour (£9/hour) for completing the survey.

## Results

### Sample Characteristics

**Figure 1:**
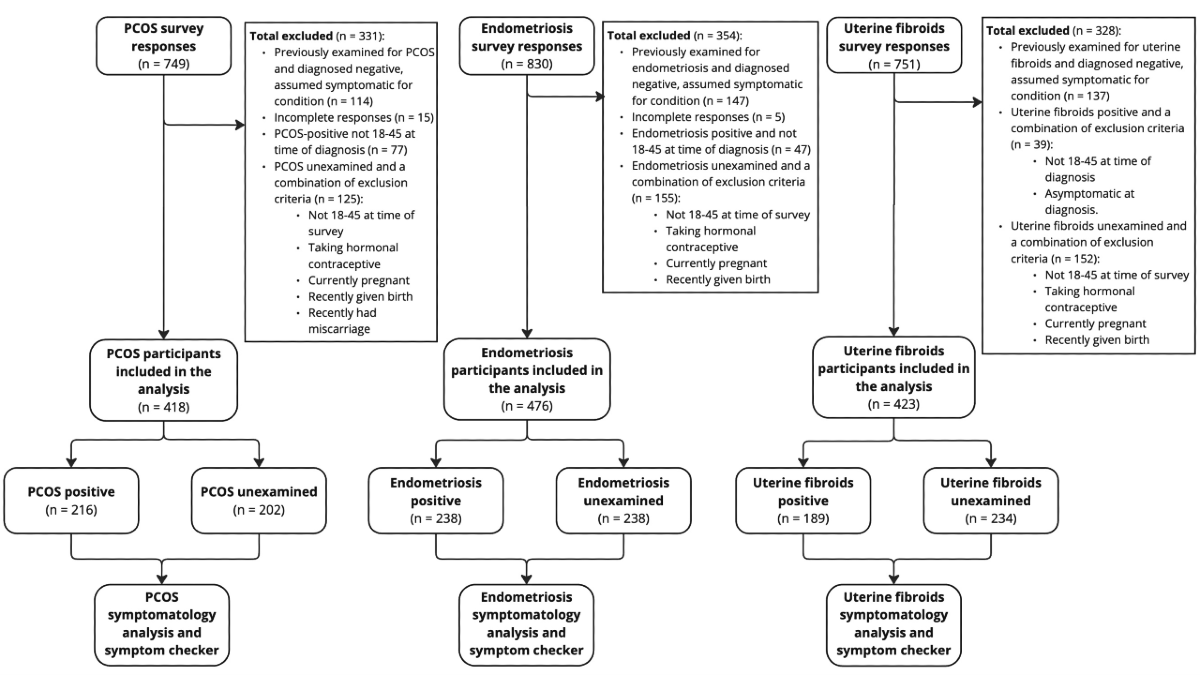
Participants flow chart. A total of 749 polycystic ovary syndrome (PCOS), 830 endometriosis, and 751 uterine fibroids symptom survey responses were collected from September to December, 2023 and filtered according to the exclusion criteria. In total, 216 PCOS-positive and 202 PCOS unexamined, 238 endometriosis-positive and 238 endometrioses unexamined, and 189 uterine fibroids-positive and 234 uterine fibroids unexamined were taken forward for analysis as part of an exploratory study investigating symptomatology and symptom checker evaluation.

A total of 2330 survey responses were collected, with 749, 830, and 751 responses collected for PCOS, endometriosis, and uterine fibroids, respectively (Figure 1). After exclusion, we collected 216 and 238 responses that had a positive diagnosis for PCOS and endometriosis, and 189 responses that had a positive, symptomatic diagnosis for uterine fibroids that we determined as our CP cases. From CN cases, those who had not been examined for the condition, we included responses from 202 participants for PCOS, 238 for endometriosis, and 234 for uterine fibroids.

The CP mean age at diagnosis was 26.6 (SD 5.7), 28.6 (SD 6.3), and 33.4 (SD 6.8) years for PCOS, endometriosis, and uterine fibroids, respectively (Table S1 in Multimedia Appendix 1). The CP group for uterine fibroids had the highest share of Black people (19.6%), compared with PCOS (8.8%) and endometriosis (8.0%; Table S1 in Multimedia Appendix 1). Among CP groups, the rate of condition-family history was similar for PCOS and endometriosis (28.7% and 33.6%, respectively) and higher for the uterine fibroids group (45.0%). Among CN groups, the rate of condition-family history for PCOS, endometriosis, and uterine fibroids was similar at 10.9%, 10.1%, and 12%, respectively.

### Self-Reported Symptomatology

**Figure 2:**
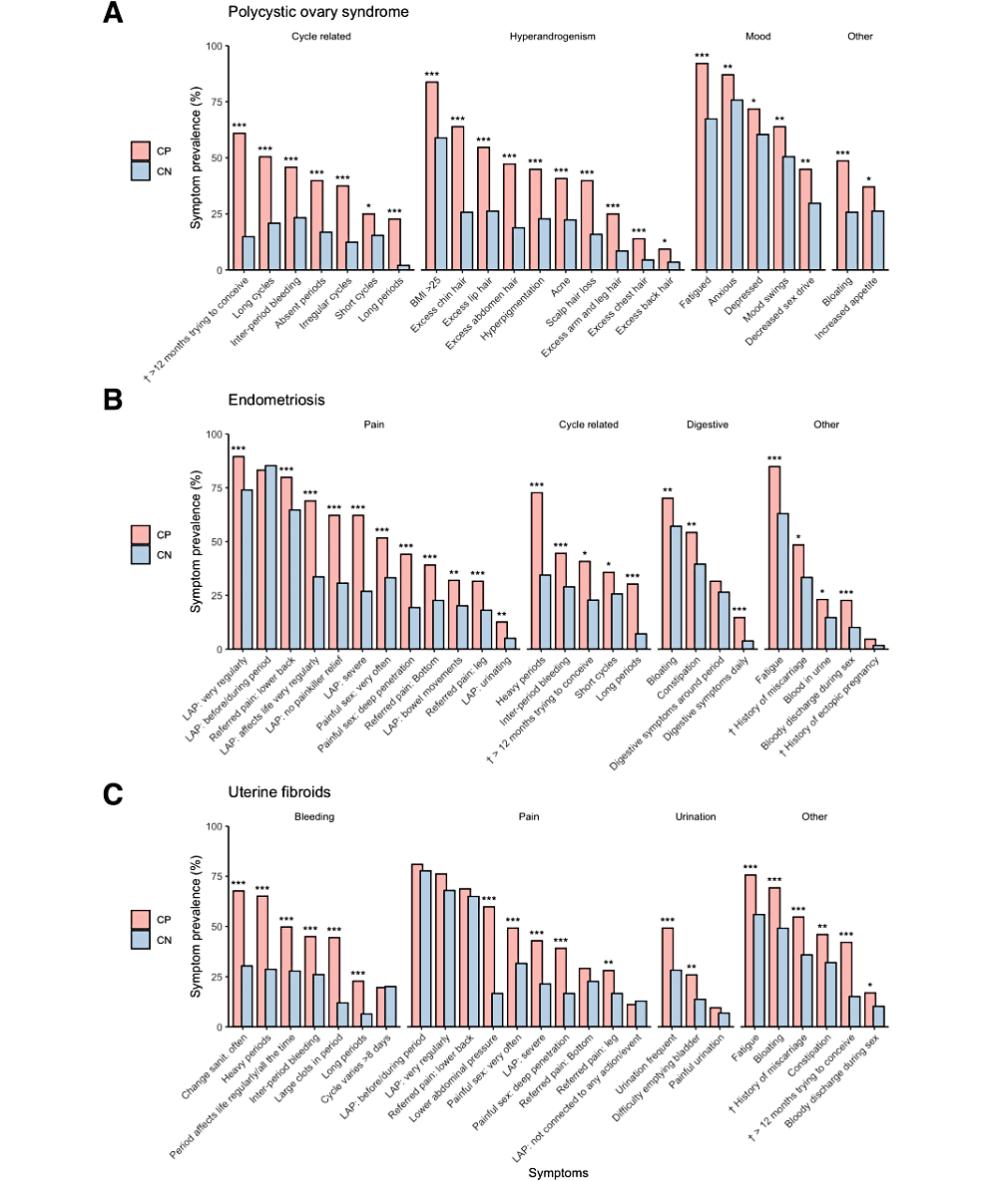
Self-reported symptomatology for condition-positive and condition-negative participants for (A) polycystic ovary syndrome, (B) endometriosis, and (C) uterine fibroids. Symptoms were self-reported and in response to survey questions from Flo single-condition symptom checkers. Statistical significance between groups is indicated: *P<.05; **P<.01; ***P<.001. † symptom prevalence is reported for those who have previously tried or are currently trying to conceive. LAP: Lower abdominal pain.

All included symptoms were significantly more prevalent among PCOS-positive compared with PCOS-negative participants (Figure 2A and Table S2A in Multimedia Appendix 1) with “Fatigue” (92%) being the most prevalent. “Excess chin hair” was reported considerably more by the PCOS-positive group (64%) than within the PCOS-negative group (26%). “BMI over 25” was highly prevalent in the PCOS-positive group (84%) and was also reported among the majority (59%) in the PCOS-negative group. PCOS-positive participants reported absent periods (amenorrhea) and irregular cycles 40% and 38% of the time, respectively.

The most prevalent symptoms reported by endometriosis-positive participants, with a significant difference to endometriosis-negative participants, were “Lower abdominal pain: very regularly” (89%), “Fatigue” (85%), and “Referred pain: lower back” (80%; Figure 2B and Table S3B in Multimedia Appendix 1). “Lower abdominal pain before or during period” (dysmenorrhea) was highly prevalent in both the endometriosis-positive and negative groups (83% and 85%) with no significant difference between them (*P*=.53). Pain that affects the participant’s life regularly (“Lower abdominal pain: affects life very regularly”) was much more prevalent in the endometriosis-positive group compared with the negative group (69% and 34%).

The most prevalent symptoms reported by uterine fibroids-positive participants, with a significant difference to uterine fibroids-negative respondents, were “Fatigue” (76%), “Bloating” (69%), and “Changing sanitary protection often” (68%; Figure 2C and Table S3C in Multimedia Appendix 1). “Lower abdominal pain before or during period” was the most prevalent symptom in both the uterine fibroids-positive and negative groups (81% and 73%) with no statistical significance between them (*P*=.42). “Lower abdominal pressure” was the most prevalent pain symptom amongst uterine fibroids-positive participants (60%) with a large difference compared with the negative group (17%).

Amongst participants with a current or previous history of trying to conceive, 60% of PCOS-positive participants, 41% of endometriosis-positive participants, and 42% of uterine fibroids-positive participants reported they were trying or had tried to conceive for 12 months or longer compared with 15%, 23%, and 15% of PCOS-, endometriosis- and uterine fibroids-negative groups, respectively. Amongst the same group of participants, a history of miscarriage was reported at 48% and 55% for endometriosis- and uterine fibroids-positive participants compared with 33% and 36% for endometriosis- and uterine fibroids-negative participants.

A sensitivity analysis investigating symptomatology of participants with a confirmed negative diagnosis (after examination) for each condition found this group had a higher prevalence of nearly all symptoms compared with the CN group used in our main analysis (Figure S1 in Multimedia Appendix 1).

### Symptom Importance

The 10 most predictive symptoms of each condition identified by LASSO regression are presented in Table 1. Interestingly for PCOS, certain locations of hirsutism (abdomen and chin) were identified as important, indicating the self-reporting of other hirsutism locations were not as predictive of the condition. For endometriosis and uterine fibroids, many shared symptoms (ie, painful sex, heavy periods, and complications with fertility) have been identified as important for condition prediction.

**Table 1:** Top 10 most predictive symptoms for identifying polycystic ovary syndrome, endometriosis, and uterine fibroids, determined by least absolute shrinkage and selection operator regression. Symptoms were self-reported and in response to survey questions from Flo single-condition symptom checkers from condition-positive and condition-negative participants.

PCOS^a^		Endometriosis	Uterine fibroids
**Cycle**			
	Long periods	—^b^	—
	Absent periods	—	—
	Cycle varies 8 days	—	—
	Interperiod bleeding	—	—
**Pain**			
	—	Severe lower abdominal pain	Lower abdominal pressure
	—	Painful sex: deep penetration	Painful sex: deep penetration
	—	Pain affects life regularly/always	—
	—	No relief from painkillers	—
**Bleeding**			
	—	Heavy periods	Heavy periods
	—	Long periods	Long periods
	—	—	Change tampon/pad regularly
	—	—	Period clots
**Hyperandrogenism**			
	Excess chin hair	—	—
	Excess abdominal hair	—	—
	Scalp hair loss	—	—
	BMI>25	—	—
**Fertility**			
	—	Trying to conceive >12 months	Trying to conceive >12 months
	—	History of miscarriage	History of miscarriage
**Bladder control**			
	—	—	Frequent urination
	—	—	Difficulty emptying bladder
**Other**			
	Fatigue	Fatigue	—
	Bloating	Daily digestive symptoms	—

^a^PCOS: polycystic ovary syndrome.

^b^—: not applicable.

### Symptom Checker Accuracy

For symptom checker performance, we found an accuracy of 78% for PCOS, 73% for endometriosis, and 75% for uterine fibroids (Table 2). Sensitivity and specificity were 76% and 81% for PCOS, 73% and 73% for endometriosis, and 78% and 73% for uterine fibroids.

The sensitivity analysis considering participants with a confirmed negative diagnosis for the condition found that accuracy reduced slightly due to a decrease in specificity (Table S3 in Multimedia Appendix 1).

**Table 2:** Performance metrics for polycystic ovary syndrome, endometriosis, and uterine fibroids symptom checkers. Symptoms used in symptom checker evaluation were self-reported and in responses to survey questions containing the same questions as the Flo symptom checkers. Symptom checker performance was evaluated when classifying between condition-positive and condition-negative cases as either “significant match” or “low or no match.”

Metric	PCOS^a^	Endometriosis	Uterine fibroids
CP^b^, n	216	238	189
CN^c^, n	202	238	234
Accuracy (%)	78	73	75
Sensitivity (%)	76	73	78
Specificity (%)	81	73	73
PPV^d^ (%)	81	73	70
NPV^e^ (%)	76	73	81

^a^PCOS: polycystic ovary syndrome.

^b^CP: condition positive.

^c^CN: condition negative.

^d^PPV: positive predictive value.

^e^NPV: negative predictive value.

## Discussion

### Preliminary Findings

In this exploratory study, we created and distributed surveys based on Flo’s symptom checkers for PCOS, endometriosis, and uterine fibroids. We collected self-reported symptoms, analyzed their prevalence and predictive value for each condition, and evaluated the symptom checker’s performance. For each condition, the top 3 most prevalent symptoms with a statistically significant difference between the CP and CN group were as follows: for PCOS-positive, “Fatigue” (92%, *P*<.001), “Anxious” (87%, *P*=.003), and “BMI over 25” (84%, *P*<.001), for endometriosis-positive, “Lower abdominal pain: very regularly” (89%, *P*<.001), “Fatigue” (85%, *P*<.001), and “Referred pain: lower back” (80%, *P*<.001); for uterine fibroids-positive, “Fatigue” (76%, *P*<.001), “Bloated” (69%, *P*<.001), “Change sanitary products often” (68%, *P*<.001).

There was no statistical difference in prevalence for “Lower abdominal pain: before or during period,” “Lower abdominal pain: very regularly,” and “Referred pain: lower back” between CP and CN groups for endometriosis and uterine fibroids participants, despite these symptoms being commonly associated with both conditions. For some symptoms, such as “Fatigue,” “Anxious,” “Lower abdominal pain: very regularly,” despite the difference in prevalence between CP and CN groups being statistically significant, the prevalence in the CN group was still high. These findings suggest that some symptoms that are clinically recognized as being associated with these conditions in medical guidelines are commonly self-reported by healthy people as well.

For PCOS, LASSO regression identified particular symptoms related to cycle irregularity (varied cycle length, long periods, and absent periods) and hyperandrogenism as being most important for condition prediction. Interestingly, current guidelines for PCOS state that the presence of scalp hair loss and acne in isolation (without hirsutism) are weak indicators of biochemical hyperandrogenism [29], yet our analysis finds them among the most important symptoms in predicting PCOS, possibly indicating there are differences in importance between self-reported symptoms and clinical evaluation.

Endometriosis and uterine fibroids have multiple symptoms that overlap between the 2 conditions [36]. LASSO regressions identified the same symptoms related to heavy, long periods, fertility, and pain with deep penetration were identified as being important for condition prediction. The self-reported symptoms which the 2 conditions do not have in common, but were identified as predictive (ie, severe, life affecting abdominal pain for endometriosis compared with a feeling of constant abdominal pressure for uterine fibroids) may support clinical decision making and are described as such in the different guidelines. Focusing on the difference in these pain-related symptoms could give clinicians more confidence when differentiating between them. Fatigue was identified as one of the most important symptoms for the prediction of PCOS and endometriosis despite being highly prevalent in the CN groups.

We tested single-condition symptom checkers using self-reported symptom datasets and found accuracy ranged from 73%-78%. Each symptom checker performed with high sensitivity (73%-78%) and specificity (73%-81%), successfully identifying people who do and do not have the target condition to a high degree. This shows that the symptom checkers can screen people for PCOS, endometriosis, and uterine fibroids when using self-assessed, self-reported symptoms with a high level of accuracy.

### Comparison to Previous Work

Symptom-based questionnaires and screening tools do exist for reproductive health conditions such as PCOS and endometriosis. A self-assessment tool for endometriosis found sensitivity of 76% and specificity of 72%, however, the accuracy metrics are based on a relatively small number of participants (n=50) compared with our study [37]. Another study that used a questionnaire for use in PCOS diagnosis has reported 77% sensitivity and 94% specificity. However, the study surveyed women who had an existing primary complaint of infertility [38]. It is possible that this could bias performance as not all people with PCOS will experience infertility [39]. The Flo symptom checkers are designed for a broader audience who may not be experiencing fertility issues or unaware that they could have a health condition.

We previously tested the performance of Flo’s symptom checkers using vignettes (simulated patient cases) [27]. We reported the accuracy in our vignettes study to be higher (83%-88%) compared with this survey-based study. This was expected, as vignette cases were created from a panel of clinical experience, portraying classic presentations of each condition. Self-reported patient data, as collected in our study, reflect a real-world scenario with greater nuances in symptomatology on which to test our symptom checkers.

### Strengths and Limitations

Strengths of this study include the large number of CP and CN participants used to evaluate each symptom checker, likely covering extensive variations of symptomatology. This has an advantage over vignette-case studies where simulated patient cases are unlikely to represent the complexity of real patient symptomatology [40-42]. The participants used in the main analysis of this study may have multiple reproductive or other health conditions with symptom crossover, providing a complex, realistic dataset of self-reported symptoms to test Flo’s symptom checkers. Each condition survey contained questions transcribed from as they appear in Flo’s symptom checker, and their completion of surveys was completely participant led with no clinical guidance or coaching to aid answering questions or identify symptoms. Thus, the accuracy metrics for each symptom checker are likely indicative of how the Flo symptom checkers would perform once deployed.

Limitations, however, should be noted. We asked CP participants to answer questions in the survey retrospectively, to the time of their diagnosis. It is possible that self-reported symptoms from CP participants could contain bias or inaccuracies, particularly in participants who were diagnosed many years ago [40,43]. Recall bias can affect those who have been diagnosed or previously examined, resulting in participants who are more likely to report symptoms [44]. Our sensitivity analysis showed participants with a confirmed negative diagnosis generally self-reported a higher symptom prevalence for all symptoms compared with the CN group in our main analysis. This could be due to recall bias, however another explanation is that these participants sought medical examination for these conditions due to the symptoms they were experiencing. Another limitation of this study is that the CP and CN groups could have been matched more closely by age and ethnicity. These conditions can vary in prevalences across different ages and ethnicity groups, as well as the presentation of symptoms.

The CN participants used in this study had not been examined for the condition by a doctor, and it was our assumption that the large majority of this group are negative for the condition due to the relatively low symptomatic prevalence of each condition. This assumption has been used by other studies in their assessment of machine-learning tools for endometriosis prediction [45,46]. However, due to this imperfect reference category, it is likely there is a bias in the metrics reported. When refining our uterine fibroids participants, we excluded participants who said they were asymptomatic at the time of their diagnosis. The intention of this approach was to exclude participants who have asymptomatic uterine fibroids; however, it may have also removed participants who were unaware their symptoms were related to uterine fibroids.

Finally, the Flo symptom checker questions, from which we estimated the prevalence of self-reported symptomatology of each condition are based on current medical guidelines. As a result, it is possible that the symptomatology and importance of symptoms presented in this study could be biased by diagnostic constructs and entrenched clinical unknowns for each condition. Further work could investigate a larger array of self-reported general and reproductive health symptoms that extend beyond what symptoms are currently clinically accepted for each condition.

### Future Work

The evaluation of all digital health tools should follow a multistage process with increasing exposure to real environments [25]. The next step in evaluating Flo symptom checkers is early-field testing; using real-world data collected from users using the symptom checker, and comparing the symptom checker’s output to the user’s official diagnosis they received from their doctor. The symptom checker performance will be continually reviewed and adjusted using the most up to date evidence and guidelines as part of an iterative product development process to ensure its safety and accuracy.

Symptom checkers that provide accurate screening of reproductive conditions could help facilitate conversations with health care providers and assist in clinical evaluation, reducing the time to diagnosis and decreasing the risk of complications caused from untreated conditions that drive up health care costs [47-49]. Access to health tools, such as symptom checkers, for conditions like PCOS, endometriosis, and uterine fibroids holds the potential to be a cost-effective strategy for reducing this economic burden, especially when evaluation represents a fraction of the all-cost total [50], and warrants further study. Future work should investigate the potential economic impact of the symptom checkers and the cost-saving potentials to health care systems and economies.

### Conclusions

Characterization of reproductive conditions by self-reported symptoms could lead to more effective screening and is vital when developing digital health solutions such as symptom checkers. In this exploratory study, we have presented the self-reported symptomatology for PCOS, endometriosis, and uterine fibroids, and analyzed the importance of each self-reported symptom when identifying each condition. Under these testing conditions, the Flo symptom checkers show high levels of accuracy, sensitivity, and specificity when assessing real, self-reported symptoms. Innovative health app solutions hold the potential to help people seek treatment before further morbidities and health complications occur.

## Appendix

Multimedia Appendix 1 Supplementary materials.

## Abbreviations

CN: condition-negative
CP: condition-positive
LASSO: Least absolute shrinkage and selection operator
PCOS: polycystic ovary syndrome

## Data Availability

Due to the sensitivity of the data subject (ie, reproductive health symptom data), we have restricted the availability of the data. The data used for this study can be made available in aggregated format through reasonable requests to the corresponding author.
