# Supplementary material for: Exploring Self-Reported Symptoms for Developing and Evaluating Digital Symptom Checkers for Polycystic Ovarian Syndrome, Endometriosis, and Uterine Fibroids: Exploratory Survey Study
Source: JMIR Form Res. 2024 Dec 12;8:e65469. doi: 10.2196/65469 (PMC11672639; doi:10.2196/65469)
Supplement: Multimedia Appendix 1 [file formative_v8i1e65469_app1.docx]

# Supplementary Materials

**Supplementary Table 1:** Sample characteristics of condition-positive and condition-negative participants.

*Age is reported as age at diagnosis for CP respondents, and age at time of survey for CN respondents

|  | | N | Age* (sd) | Ethnicity | | | |
| --- | --- | --- | --- | --- | --- | --- | --- |
|  | |  |  | White (%) | Black (%) | Asian (%) | Mixed (%) |
| PCOS | |  |  |  |  |  |  |
|  | Condition positive | 216 | 26.6 (5.7) | 72.7 | 8.8 | 4.6 | 8.3 |
|  | Condition negative | 202 | 31.7 (6.8) | 63.3 | 11.4 | 10.9 | 9.4 |
| Endometriosis | |  |  |  |  |  |  |
|  | Condition positive | 238 | 28.6 (6.3) | 81.0 | 8.0 | 1.3 | 6.3 |
|  | Condition negative | 238 | 31.4 (6.9) | 63.4 | 12.1 | 12.6 | 7.6 |
| Uterine Fibroids | |  |  |  |  |  |  |
|  | Condition positive | 189 | 33.4 (6.8) | 67.7 | 19.6 | 2.1 | 7.4 |
|  | Condition negative | 234 | 31.1 (6.9) | 66.3 | 10.2 | 11.9 | 7.7 |

**Supplementary Table 2:** Symptom prevalences of (A) polycystic ovary syndrome (B) endometriosis and (C) uterine fibroids, for condition-positive (CP) and condition-negative (CN) participants. P-values are calculated using Chi-square tests without correction.

† Prevalences calculated for those who indicated they have previously tried or are currently trying to conceive, only.

LAP: Lower abdominal pain

| **A) Polycystic Ovary Syndrome** | | | | |
| --- | --- | --- | --- | --- |
| **Group** | **Symptom** | **Prevalence (CP)** | **Prevalence (CN)** | **p** |
| Mood | Fatigued | 0.92 | 0.67 | p<0.001 |
| Mood | Anxious | 0.87 | 0.76 | 0.003 |
| Hyperandrogenism | BMI >25 | 0.84 | 0.59 | p<0.001 |
| Mood | Depressed | 0.72 | 0.60 | 0.014 |
| Hyperandrogenism | Excess chin hair | 0.64 | 0.26 | p<0.001 |
| Mood | Mood swings | 0.64 | 0.50 | 0.006 |
| Cycle related | † >12 months trying to conceive | 0.61 | 0.15 | p<0.001 |
| Hyperandrogenism | Excess lip hair | 0.55 | 0.26 | 〃 |
| Cycle related | Long cycles | 0.50 | 0.21 | 〃 |
| Other | Bloating | 0.49 | 0.26 | 〃 |
| Hyperandrogenism | Excess abdomen hair | 0.47 | 0.19 | 〃 |
| Cycle related | Inter-period bleeding | 0.46 | 0.23 | 〃 |
| Hyperandrogenism | Hyperpigmentation | 0.45 | 0.23 | 〃 |
| Mood | Decreased sex drive | 0.45 | 0.30 | 0.001 |
| Hyperandrogenism | Acne | 0.41 | 0.22 | 〃 |
| Cycle related | Absent periods | 0.40 | 0.17 | 〃 |
| Hyperandrogenism | Scalp hair loss | 0.40 | 0.16 | 〃 |
| Cycle related | Irregular cycles | 0.38 | 0.12 | 〃 |
| Other | Increased appetite | 0.37 | 0.26 | 0.018 |
| Cycle related | Short cycles | 0.25 | 0.15 | 0.014 |
| Hyperandrogenism | Excess arm and leg hair | 0.25 | 0.08 | p<0.001 |
| Cycle related | Long periods | 0.23 | 0.02 | 〃 |
| Hyperandrogenism | Excess chest hair | 0.14 | 0.04 | 〃 |
| Hyperandrogenism | Excess back hair | 0.09 | 0.03 | 0.016 |
| **B) Endometriosis** | | | | |
| **Group** | **Symptom** | **Prevalence (CP)** | **Prevalence (CN)** | **p** |
| Pain | LAP: very regularly | 0.89 | 0.74 | p<0.001 |
| Other | Fatigue | 0.85 | 0.63 | 〃 |
| Pain | LAP: before/during period | 0.83 | 0.85 | 0.529 |
| Pain | Referred pain: lower back | 0.80 | 0.65 | p<0.001 |
| Cycle related | Heavy periods | 0.73 | 0.34 | 〃 |
| Digestive | Bloating | 0.70 | 0.57 | 0.003 |
| Pain | LAP: affects life very regularly | 0.69 | 0.34 | p<0.001 |
| Pain | LAP: no painkiller relief | 0.62 | 0.31 | 〃 |
| Pain | LAP: severe | 0.62 | 0.27 | 〃 |
| Digestive | Constipation | 0.54 | 0.39 | 0.001 |
| Pain | Painful sex: very often | 0.52 | 0.33 | p<0.001 |
| Other | † History of miscarriage | 0.48 | 0.33 | 0.033 |
| Cycle related | Inter-period bleeding | 0.45 | 0.29 | p<0.001 |
| Pain | Painful sex: deep penetration | 0.44 | 0.19 | 〃 |
| Cycle related | † > 12 months trying to conceive | 0.41 | 0.23 | 0.018 |
| Pain | Referred pain: Bottom | 0.39 | 0.23 | p<0.001 |
| Cycle related | Short cycles | 0.36 | 0.26 | 0.017 |
| Pain | LAP: bowel movements | 0.32 | 0.20 | 0.003 |
| Pain | Referred pain: leg | 0.32 | 0.18 | p<0.001 |
| Digestive | Digestive symptoms around period | 0.32 | 0.26 | 0.225 |
| Cycle related | Long periods | 0.30 | 0.07 | p<0.001 |
| Other | Blood in urine | 0.23 | 0.15 | 0.019 |
| Other | Bloody discharge during sex | 0.23 | 0.10 | p<0.001 |
| Digestive | Digestive symptoms daily | 0.15 | 0.04 | 〃 |
| Pain | LAP: urinating | 0.13 | 0.05 | 0.004 |
| Other | † History of ectopic pregnancy | 0.05 | 0.02 | 0.455 |
| **C) Uterine fibroids** | | | | |
| **Group** | **Symptom** | **Prevalence (CP)** | **Prevalence (CN)** | **p** |
| Pain | LAP: before/during period | 0.81 | 0.78 | 0.424 |
| Pain | LAP: very regularly | 0.76 | 0.68 | 0.062 |
| Other | Fatigue | 0.76 | 0.56 | p<0.001 |
| Other | Bloating | 0.69 | 0.49 | 〃 |
| Pain | Referred pain: lower back | 0.69 | 0.65 | 0.407 |
| Bleeding | Change sanit. often | 0.68 | 0.30 | p<0.001 |
| Bleeding | Heavy periods | 0.65 | 0.29 | 〃 |
| Pain | Lower abdominal pressure | 0.60 | 0.17 | 〃 |
| Other | † History of miscarriage | 0.55 | 0.36 | 〃 |
| Bleeding | Period affects life regularly/all the time | 0.50 | 0.28 | 〃 |
| Pain | Painful sex: very often | 0.49 | 0.32 | 〃 |
| Urination | Urination frequent | 0.49 | 0.28 | 〃 |
| Other | Constipation | 0.46 | 0.32 | 0.003 |
| Bleeding | Inter-period bleeding | 0.45 | 0.26 | p<0.001 |
| Bleeding | Large clots in period | 0.44 | 0.12 | 〃 |
| Pain | LAP: severe | 0.43 | 0.21 | 〃 |
| Other | † > 12 months trying to conceive | 0.42 | 0.15 | 〃 |
| Pain | Painful sex: deep penetration | 0.39 | 0.17 | 〃 |
| Pain | Referred pain: Bottom | 0.29 | 0.23 | 0.130 |
| Pain | Referred pain: leg | 0.28 | 0.17 | 0.005 |
| Urination | Difficulty emptying bladder | 0.26 | 0.14 | 0.001 |
| Bleeding | Long periods | 0.23 | 0.06 | p<0.001 |
| Bleeding | Cycle varies >8 days | 0.20 | 0.20 | 0.896 |
| Other | Bloody discharge during sex | 0.17 | 0.10 | 0.044 |
| Pain | LAP: not connected to any action/event | 0.11 | 0.13 | 0.591 |
| Urination | Painful urination | 0.10 | 0.07 | 0.312 |


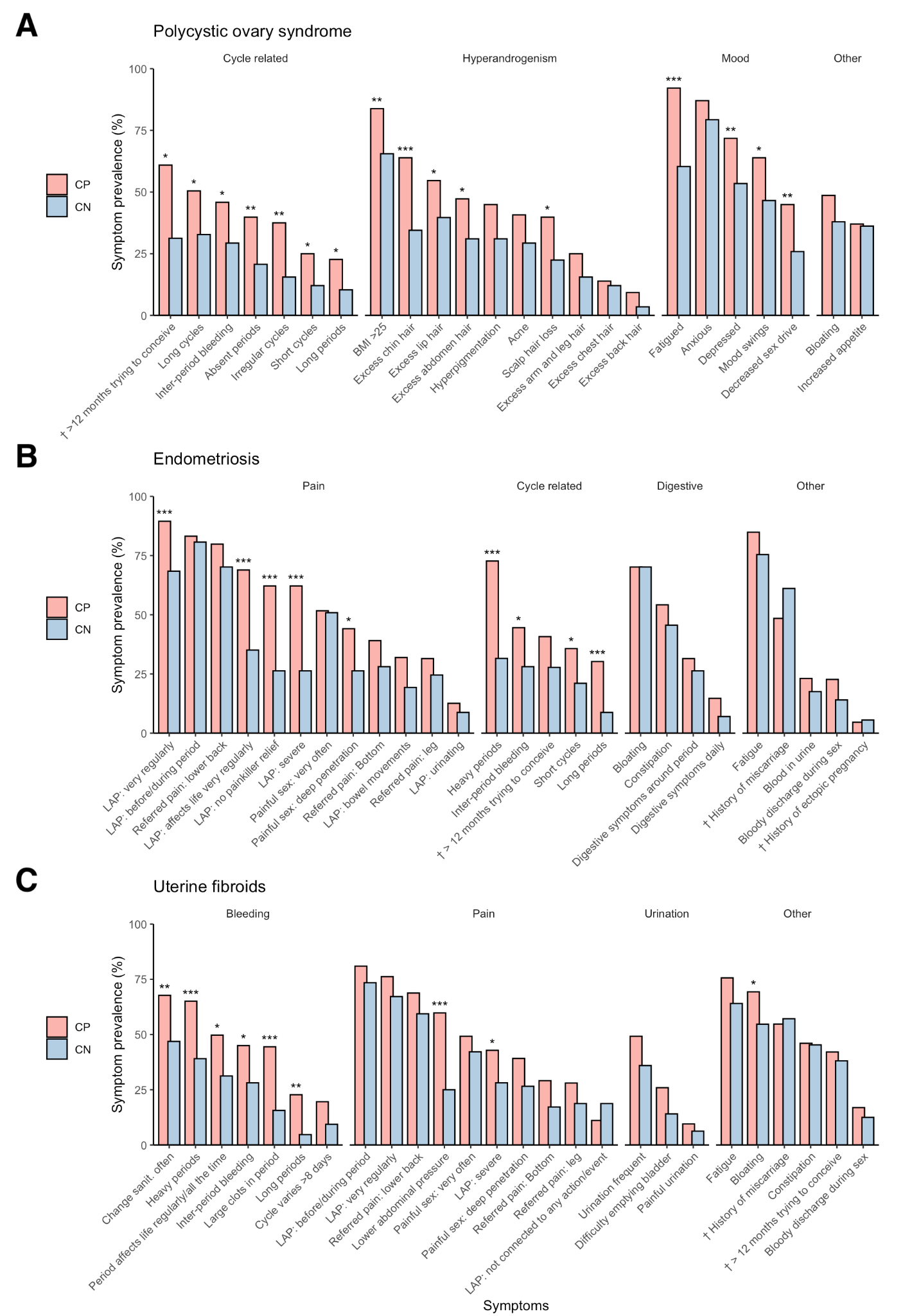


**Supplementary Figure 1:** Condition symptomatology for condition-positive (CP) and participants who have been examined and confirmed to be condition-negative (CN). Symptoms are self-reported and in response to questions from Flo single-condition symptom checkers. CN groups are individuals who have been examined for the condition and told they do not have it. Statistical significance between groups is indicated: * *P* < 0.05; ** *P* < 0.01; *** *P* < 0.001. LAP: Lower abdominal pain.

**Supplementary Table 3:** Accuracy metrics for polycystic ovary syndrome, endometriosis, and uterine fibroids symptom checkers when comparing condition-positive and confirmed negative (those examined for the condition and told they do not have it) groups.

| Metric | PCOS^a^ | Endometriosis | Uterine fibroids |
| --- | --- | --- | --- |
| N_CP_^d^ | 216 | 238 | 189 |
| N_confirmed negative_ | 58 | 57 | 64 |
| Accuracy | 73 | 72 | 74 |
| Sensitivity | 76 | 73 | 78 |
| Specificity | 64 | 68 | 62 |
| PPV^b^ | 89 | 91 | 86 |
| NPV^c^ | 42 | 38 | 49 |

^a^PCOS: polycystic ovary syndrome.

^b^PPV: positive predictive value

^c^NPV: negative predictive value

^d^CP: condition positive
